# A comprehensive protocol to study the effects of multilingualism on cognition and the brain in patients with progressive neurological diseases

**DOI:** 10.1016/j.mex.2025.103343

**Published:** 2025-04-29

**Authors:** Christos Pliatsikas, Mark Antoniou, Jon Andoni Duñabeitia, Marco Calabria

**Affiliations:** a School of Psychology and Clinical Language Studies, University of Reading, UK; b Centro de Investigación Nebrija en Cognición, Universidad Nebrija, Madrid, Spain; c The MARCS Institute for Brain, Behaviour and Development, Western Sydney University, Australia; d Faculty of Health Sciences, Universitat Oberta de Catalunya, Barcelona, Spain

**Keywords:** Bilingualism, Multilingualism, Cognitive reserve, Neuroimaging, Parkinson’s, Huntington’s, Alzheimer’s, Multiple Sclerosis

## Abstract

Multilingualism, the cognitively demanding experience of speaking multiple languages, has been linked to measurable effects on cognition and brain structure and function. These effects, attributed to the constant activation of multiple languages, may contribute to cognitive reserve, expressed as delayed cognitive decline in multilinguals diagnosed with progressive neurological diseases such as Alzheimer’s, Huntington’s and Parkinson’s diseases, and Multiple Sclerosis. However, the evidence remains inconsistent due to methodological shortcomings, including variations in defining multilingualism and inadequate accounting for other lifestyle factors that may also promote cognitive reserve. To address these limitations, we developed a comprehensive protocol which incorporates detailed measurement of factors that are related to cognitive reserve, including multilingual experiences, standardized clinical and experimental cognitive assessments suitable for multilingual patients diagnosed with the above progressive neurological diseases, as well as thorough neuroimaging testing batteries. This protocol enables systematic investigation across linguistic and cultural contexts, facilitating cross-laboratory data pooling to advance understanding of multilingualism’s neuroprotective potential in aging and disease. Its purpose is to spearhead an open-ended project which will keep adding to an increasing dataset of multilingual patients internationally; crucially, it sets a new standard in how to gather evidence investigating multilingualism as a factor contributing to cognitive reserve.

## Graphical abstract

**Figure:**
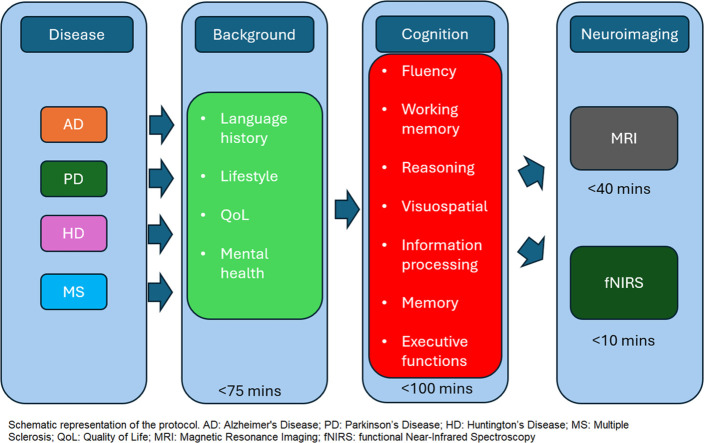

Specifications table

**Table:** 

Subject area:	Neuroscience
More specific subject area:	Cognitive neuroscience of multilingualism and ageing
Name of your protocol:	MULTIDEGEN
Reagents/tools:	N/A
Experimental design:	A comprehensive protocol administering a series of behavioural and neuroimaging experiments to assess how multilingualism interacts with the impact of progressive neurological diseases on cognition and the brain.
Trial registration:	N/A
Ethics:	This is not applicable because this is a prospective protocol that has not been applied yet. Nevertheless, no major ethical issues are envisaged. The behavioural and neuroimaging batteries are widely used in research with patients with progressive neurological diseases. Data will be anonymised at the point of collection, with participant numbers assigned in order to assist with linking the different types of data for the purposes of the analysis. MRI images will be defaced as part of preprocessing, and the original images will be discarded. The ethical protocols of the participating universities and hospitals will be adhered to.
Value of the Protocol:	• The first comprehensive protocol of its kind • Appropriate for use in various linguistic and cultural contexts • It allows for multi-lab collaborations for data collection and analysis

## Background

The cognitively challenging experience of speaking more than one language (bi-/multilingualism, henceforth simply “multilingualism”) has been shown to have measurable effects on cognition and brain structure and function. These effects typically manifest as better performance of multilinguals vs. monolinguals in several cognitive tasks, as well as differences in structure, function and connectivity of brain regions underlying language processing and control [[1], [2], [3]]. These adaptations have been attributed to the well-documented concurrent activation of all spoken languages, which requires heightened levels of attentional control [4]. These increased cognitive demands have been proposed to alter the underlying brain networks in order to lead to more efficient language control, indications of which may even be found in non-linguistic tasks [5]. Although the evidence remains inconsistent [6], the observed discrepancies have been attributed to various methodological issues, including, crucially, how multilingualism is defined and how the language experiences of the multilinguals are measured and accounted for [[7], [8], [9]].

Crucially, effects of multilingualism have been observed in patients with progressive neurological diseases including Alzheimer’s Disease (AD) and, to a much smaller extent, Huntington’s Disease (HD) and Multiple Sclerosis (MS), whereas evidence from Parkinson’s Disease (PD) remains unclear [10]. These effects have manifested as slower rate of neurodegeneration and/or better preserved cognition for longer periods of time, when compared to monolingual patients. This has led to suggestions that multilingualism may counteract the effects of brain decline in patients with neurological conditions, by contributing towards building a *cognitive reserve*, defined as “the discrepancy between the observed and expected level of cognitive ability, given the observed level of neural integrity” [11]. This echoes similar suggestions for other types of cognitively enriching activities such as education, occupation, exercise, musical training etc., which have also been suggested to contributed towards cognitive and brain reserves [12,13].

The literature on the neuroprotective effects of cognitively stimulating experiences on these diseases remains inconclusive [[14], [15], [16], [17]]. This is particularly evident in the case of multilingualism, where evidence remains limited due to small sample sizes, heterogeneous participant characteristics, and inconsistencies in testing protocols across studies. This is especially striking given that multilingualism is a highly prevalent phenomenon [18] and a common requirement in many communities around the world—unlike leisure activities such as exercise. Thus, a systematic investigation of the effects of multilingualism on the declining brain emerges as timely and imperative.

Following up from our previous work [[19], [20], [21], [22], [23], [24]], we present a new behavioural and neuroimaging protocol which brings together a variety of established tools assessing language and other life experiences, brain function and structure, and cognition. The protocol allows for the quantification of life experiences that affect the declining brain, including multilingualism; crucially, the cognitive assessment combines standardized clinical tests and experimental tasks that are methodologically sound and suitable for testing multilingual patients of the four major neurological diseases of interest (AD, PD, HD, MS). Finally, the included tools are readily applicable to, and compatible with, different linguistic and cultural contexts, which will allow for the eventual pooling of data from different labs.

## Description of protocol

### Participants

This protocol maximally allows for the recruitment of participants from participating research groups globally. This is imperative given the relative scarcity of bi-/multilingual patients of the target diseases, and the challenges linked to conducting research with such samples [10,25]. In terms of targeted sample sizes, based on previous research that used the same approach as ours on older bilingual participants [23,24,26], a minimum sample size of 100 patients per target disease will be pursued, which we consider to be feasible within about 5 years.

Validated clinical diagnostic criteria will be used for each disease type:

• AD: The recommendations from the National Institute on Aging-Alzheimer’s Association workgroups on diagnostic guidelines [27] or the revised criteria for diagnosis and staging [28]
• PD: The Movement Disorder Society’s clinical diagnostic criteria [29], in alignment with disease severity assessments using the Hoehn and Yahr Scale or the Unified Parkinson’s Disease Rating Scale [30], if available.
• HD: The criteria proposed by Reilmann and colleagues [31] or the revised version by Considine and colleagues [32], along with the Unified Huntington's Disease Rating Scale (UHDRS) [33] score for disease severity, if available.
• MS: The revised McDonald criteria [34].

Patients with potentially confounding neurological (other than those of interest) and psychiatric disorders, clinically diagnosed hearing or vision impairments that may compromise their responses in different tasks, a history of alcohol abuse, and/or psychosis will be excluded from the study.

The type of ongoing pharmacological treatment at the time of recruitment is not an inclusion or exclusion criterion; however, this information will be collected for data analysis, including the duration of drug therapy and any changes that occur during testing.

### Protocol administration

All the questionnaires and behavioural tasks described below will be administered online via the Gorilla platform [35]. Third-party tools that we will not be able to programme ourselves (e.g. the Language History Questionnaire, see below) will link from the Gorilla platform. This approach will facilitate widespread but centralised and standardised data collection with minimal costs, following from our recent practice [36]. Participating labs will need to cover the cost of participant recruitment and testing.

### Background questionnaires, to be administered online at any time before the behavioural and neuroimaging testing (approximately 75 min)

#### Language background information

The Language History Questionnaire 3.0 (LHQ3) [37] will be used to collect detailed information about the participants’ **linguistic background**, including patterns of use and switching between multiple languages. The LHQ3 is available in online and pen-and-paper formats, and it takes 20–30 min to administer. It comprises 27 questions which are used to calculate aggregate scores assessing overall proficiency, dominance, and immersion levels of each of the language that participants have learnt, as well as a Multilingual Language Diversity (MLD) score, which captures the overall linguistic experiences of multilinguals. The MLD is based on the concept of language entropy [38], and is increasingly used as a predictor of bilingualism-induced brain changes [26]. The LHQ3 is currently available in 43 languages, and it allows for translations in more languages as needed.

#### Lifestyle factors

Our protocol will include the Cognitive Reserve Index questionnaire (CRIq) [39], a widely used tool assessing educational level as well as frequency of involvement in **cognitively stimulating activities** such as working activities and leisure time activities. It comprises 20 questions and takes up to 15 min to administer. It can then be used to calculate three subscores for education, working activity, and leisure time activities, and an overall CRI score which has already been used in studies looking at the effects of such activities on brain and cognition [40]. CRIq is currently available in 16 languages, and more translations can be added as appropriate.

Additionally, and following from the recommendations of the recent *Lancet* report on dementia prevention, intervention and care [41], several **key factors linked to dementia progression** will be measured. Specifically, participants will be asked about their (a) alcohol consumption, measured as self-reported total units of alcohol consumed on a weekly basis over the previous year [42]; (b) smoking habits, to classify them as either current/past smokers or non-smokers [42]; (c) physical activity (days per week, duration of exercise, type of activities (e.g., walking, running, weightlifting), and intensity (light, moderate, vigorous); (d) Body Mass Index (BMI), which is calculated by dividing a person’s weight in kilograms by the square of their height in meters, providing a simple, widely used indicator for assessing obesity risk and overall body fat; (e) Waist Circumference (WC), measured using a flexible, non-stretchable tape placed snugly around the bare abdomen—typically at the midpoint between the lowest rib and the iliac crest—while the person stands upright and exhales gently. This can be used to evaluate cardiovascular risks (and perhaps metabolic disorders); and (f) vision and hearing impairment. Participants will also be asked to indicate the number of fruit and vegetable portions they consume on a typical day, as an indicative measure of dietary habits which is applicable to multiple cultural contexts [43]. Lists of country-specific examples of fruits and vegetables will be provided to improve response accuracy. This number will be converted into a standardised measure of fruit and vegetable consumption, expressed as 100 g/day [44]. Finally, participants will be asked to give information about any medication they take at the time of testing (5 min).

#### Quality of life and mental health

Patients’ self-perceived **quality of life** will be measured with the widely used World Health Organisation’s WHOQOL-BREF questionnaire [45]. It comprises 26 items that assess four domains of quality of life (physical, psychological, social and environment) and takes 10–15 min to administer. The WHOQOL-BREF is currently translated in 79 languages, and standardised instructions on how to translate in more languages are provided.

Moreover, the participants’ **mental health** will be assessed with the Hospital Anxiety and Depression Scale (HADS) [46]. The HADS comprises 7 items related to anxiety and 7 items related to depression and it takes 5–10 min to administer. It can be used to calculate an Anxiety score, a Depression Score, and an overall score. It is currently translated in 140 languages or language varieties, and more translations can be added.

### Cognitive assessment, to be administered in person (approximately 100 min)

Our protocol will include the Montreal Cognitive Assessment (MoCA) [47]. MoCA is a widely used cognitive screening tool designed to assess cognitive domains including memory, attention, language, visuospatial skills, and executive functions. It includes 30 items and typically takes about 10 to 15 min to administer. It produces an overall score of cognitive function. The MoCA is available in 93 languages/language varieties, and more can be added.

**Lexico-semantic word retrieval abilities** will be assessed with standard semantic and letter fluency tasks which have been extensively used with patients with neurodegenerative diseases, and have been argued to index several cognitive skills, including verbal retrieval abilities and executive functions [48]. For these tasks, participants are allowed one minute to produce as many words as they can that belong to a semantic category (semantic fluency) or start with a certain letter (letter fluency). These tasks are straightforward to administer in any language, and they will be completed in all the languages that the participants speak, using different categories/letters for each language, to avoid repetition effects. Thus, the minimum administration time would be 4 min for bilinguals. Moreover, a picture naming test will be administeredm that will use images from the Multipic dataset [36,49], which comprises 500 images with naming norms and familiarity ratings across 34 languages. We initially selected the images with the highest familiarity ratings (mean>80 %, SD<6) (*n* = 143), to ensure that the images will be familiar to patients from the widest variety of cultures. Of these, we selected the 60 images with the smallest H score, a measure of naming inconsistency, that we split into 2 groups of 30 images each (see Supplementary materials). Group 1 (Encoding items) will be presented at random, and the patients will be asked to simply name the pictures aloud in their most proficient language. The number of correct responses will be recorded. This task will last 5 min.

**Working memory** will be assessed with the backward digit span task [50], in which patients are asked to repeat backwards sequences of digits of increasing length until they fail to correctly repeat a certain sequence twice. This can easily be administered in any language, and it will be completed in all languages our patients speak. The task usually takes 5–10 min to administer, meaning that it should take about 15 min for bilingual patients.

**Non-verbal reasoning skills** will be assessed with the matrix reasoning item bank (MaRs-IB) [51], in which participants view coloured matrices of abstract shapes with a missing part, which they need to identify from an array of alternative shapes. Performance in this task (measured as accuracy, reaction times and an efficiency measure combining both) is used as an index of abstract reasoning and fluid intelligence. Since the MaRs-IB does not have a verbal component, it can be administered in any language, and the matrices are designed to be culturally neutral. It comprises 80 patterns and takes 15 min to administer.

**Visuospatial abilities** will be assessed with the Judgment of Line Orientation Test (JLO) task [52], which requires participants to judge the orientation of two lines against lines organised around a semicircular grid. It comprises 30 items and it yields an overall accuracy score. There is also no verbal element to this task, which lasts 10–20 min.

**Information processing speed** will be assessed with the Symbol Digit Modality Test (SMDT), which has been commonly used on patients with progressive neurodegeneration [53]. This test presents 9 symbols corresponding to numbers from 1–9, followed by a grid of the same symbols without numbers. Participants need to write the matching number next to as many symbols as they can within 90 s.

**Episodic memory** will be assessed with a picture recognition task. The images from Multipic that were used as encoding items in the picture naming task (Group 1) will be combined with the images from Group 2 (not presented during the encoding phase) and all 60 images will be presented to the patients at random, one at a time. The patients will simply be asked to press one of two buttons to indicate whether they had seen the image before or not, while their accuracy and reaction times will be recorded. This task will last 5–10 min and should be administered 15–20 min after the encoding phase, with a non-linguistic task in between

**Executive functions** will be assessed via a suite of tasks that have been extensively used on older bilinguals and tap inhibition, switching and updating abilities [54], i.e. skills that are important in language control in bilinguals. The tasks will be administered online, and both accuracy and reaction times will be collected. This battery will include: (a) the Flanker task (inhibition), where participants will see rows of arrows and will have to indicate the direction of the middle arrow, which may (congruent condition -easy) or may not (incongruent condition- difficult) match the direction of the flanking arrows; (b) the colour-shape task (switching), where participants will be asked to decide whether two stimuli are the same in terms of their shape or colour, and these decision would always be made on the basis of the same property (e.g. colour) (“repeat” trials- easy) or the target property would change (“switch” trials- difficult); and (c) the n-back task (updating), where participants will be presented with series of numbers and would have to indicate (yes/no) whether the number on the screen either matches a pre-specified number (e.g., “9”) (0back condition-easy), or the number presented one trial earlier (1back condition-difficult). This battery would last for about 15 min.

### MRI protocol: (40 min)

An MRI scanning protocol will be used that is appropriate for research with all of the patient groups of interest. This will include the following whole-brain sequences, acquired in a 3T scan with a 64-channel head coil:

1. A T1 MPRAGE 3D image, for the study of brain structure, especially grey matter [23] (256 sagittal slices, 0.7 mm isotropic voxels, field of view (FOV): 224  ×  224 mm, echo time (TE) = 2.41 ms, repetition time (TR)  = 2400 ms, inversion time (TI) = 1140 ms, flip angle 8°). The images will be preprocessed with standard pipelines in Freesurfer [55], to extract measures of thickness, area extent and volume of grey matter regions defined by standard atlases provided by Freesurfer.
2. A 3D Diffusion Tensor Imaging (DTI) sequence will be acquired to study water diffusivity in white matter, which is a measure of integrity and a metric of structural brain connectivity [19] (60 transverse slices, 2 mm isotropic voxels, FOV: 260 × 260 mm, TE = 70 ms, TR = 1800 ms, two averages, 64 directions, b-value: 1000 s/mm²). Standard measures of white matter integrity (Fractional Anisotropy and Mean Diffusivity) of individual white matter tracts will be estimated and analysed with Freesurfer.
3. A T2 FLAIR sequence (192 slices, voxel size: 0.4 × 0.4 × 0.9 mm, FOV: 230 × 230 mm, TE = 387 ms, TR = 5000 ms, flip angle 120°, GRAPPA acceleration Factor: 2) This will be used for the study of WM hyperintensities, which is a typical symptom of healthy ageing and diseases like multiple sclerosis [56]. FLAIR images will be analysed with a Lesion Prediction Algorithm (LPA) run through the Lesion Segmentation Tool version 2.0.15 (www.statistical-modelling.de/lst.html) [57], implemented in the SPM software [58]. This will allow for the detection, extraction and quantification of white matter hyperintensities across the entire brain.
4. A resting-state fMRI sequence, to study brain functional networks [19]. Whole-brain functional images will be acquired at rest (300 vol, 68 transversal slices, voxel size: 2.1 × 2.1 × 2.0 mm, FOV: 192 × 192 mm, TE = 30 ms, TR = 1500 ms, flip angle 66°). The data will be analysed with the Multivariate Exploratory Linear Optimized Decomposition into Independent Components (MELODIC) pipeline, part of FSL software, a data-driven approach that allows for the identification and study of functional networks [59,60]. For the purposes of this study, resting state networks that are key to cognitive control will be examined, as identified by the MELODIC atlas. These will include the frontoparietal control network (dorsolateral and inferior frontal regions and inferior parietal regions), the salience network (anterior insula, dorsal anterior cingulate gyrus and supramarginal gyri), and the default mode network (posterior cingulate gyrus, ventromedial prefrontal cortex, angular gyri and the parahippocampal gyri) [61].

### Alternative fNIRS protocol (10 mins)

A substantial proportion of the recruited participants will not be compatible with an MRI scanner because of implants, pacemakers etc. These patients will undergo neuroimaging via functional Near-Infrared Spectroscopy (fNIRS), a non-invasive method that is appropriate for studying brain function in such samples [62] but has not been systematically used in the study of multilingualism [63]. A portable system (such as MediBrite) will be used to record resting-state brain activity outside the lab and at the patients’ home or hospital. Following established methods [64], brain activity at rest will be recorded with a 27-channel set up from frontal and parietal regions bilaterally, using the 10–20 EEG system for placement. The system will employ dual-wavelength near-infrared light (760 and 850 nm) to measure oxygenated (HbO) and deoxygenated (HbR) hemoglobin at a sampling rate of 75 Hz. Optodes will be placed at a source-detector distance of 3 cm to optimize cortical signal detection. Short-separation channels (8 mm) will be included to remove extracerebral signal contributions. Participants will keep their eyes open during a 5-minute resting-state recording. Preprocessing will include motion artifact correction, band-pass filtering (0.01–0.1 Hz), and physiological noise reduction. Functional connectivity will be assessed via wavelet coherence analysis to examine inter- and intra-hemispheric connectivity patterns.

### Statistical analysis

Following up on our previous work on healthy bilinguals [20], we will analyse the data with maximal Generalised Additive Mixed Models. A series of models will include our main predictor value (MLD score) and covariates (measures of lifestyle, quality of life and mental health), separately for:

(i) Measures of cognition, expressed as accuracy and reaction times (when available) in all behavioural tasks;
(ii) Measures of regional grey matter structure (cortical thickness, surface area extent, volume), and white matter structure (Fractional Anisotropy, Mean Diffusivity) as extracted from Freesurfer;
(iii) Number and volume of white matter lesions, as estimated from LPA;
(iv) For participants undergoing resting-state fMRI, measures of resting-state activity will be averaged across each network of interest and will be used as the predicted variable;
(v) For the participants undergoing fNIRS, the predicted variables will be concentrations of HbO and HbR in each channel.

## Protocol validation

N/A

## Limitations

Not applicable.

## CRediT author statement

**Christos Pliatsikas**: Conceptualization, Methodology, Investigation, Resources, Writing - Original Draft, Writing - Review & Editing, Visualization, Project administration, Funding acquisition. **Mark Antoniou**: Methodology, Resources, Writing - Review & Editing, Project administration. **Jon Andoni Duñabeitia**: Methodology, Writing - Review & Editing. **Marco Calabria**: Methodology, Writing - Review & Editing, Funding acquisition.

**Supplementary material *and/or* additional information [OPTIONAL]**

The images from the Multipic dataset used in this protocol are provided in the supplementary materials

## Declaration of competing interest

The authors declare that they have no known competing financial interests or personal relationships that could have appeared to influence the work reported in this paper.

## Data Availability

No data was used for the research described in the article.

## Appendix Supplementary materials

Image, application 1
