# Supplementary figures and images for: A comprehensive protocol to study the effects of multilingualism on cognition and the brain in patients with progressive neurological diseases
Source: MethodsX. 2025 Apr 29;14:103343. doi: 10.1016/j.mex.2025.103343 (PMC12090328; doi:10.1016/j.mex.2025.103343)

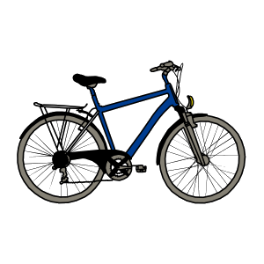

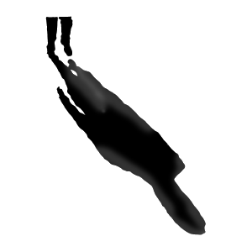

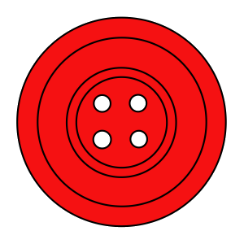

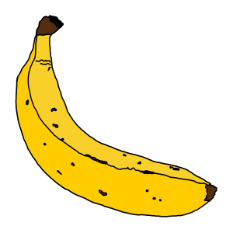

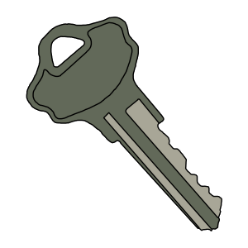


| 23 | 55 | 64 | 69 | 78 |
| --- | --- | --- | --- | --- |


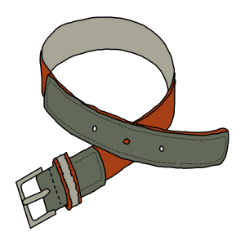

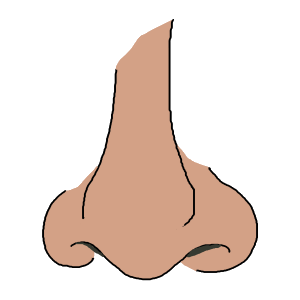

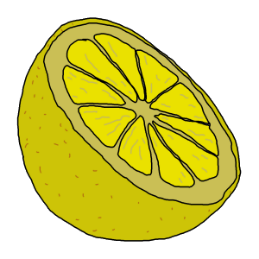

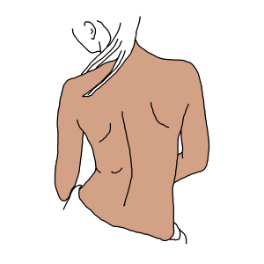

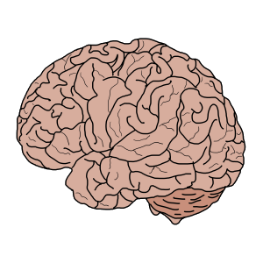


| 100 | 129 | 165 | 169 | 247 |
| --- | --- | --- | --- | --- |


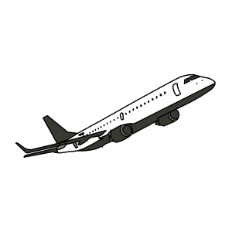

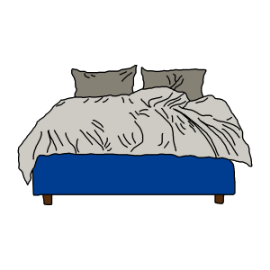

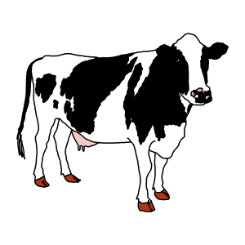

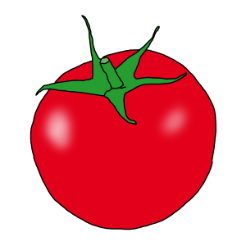

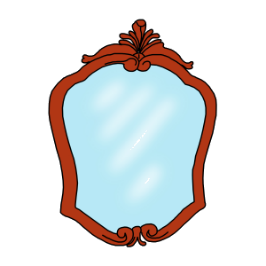


| 268 | 297 | 319 | 326 | 340 |
| --- | --- | --- | --- | --- |


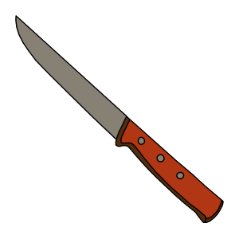

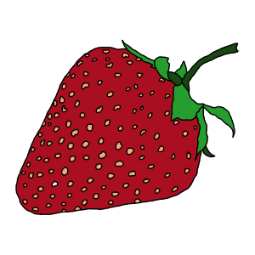

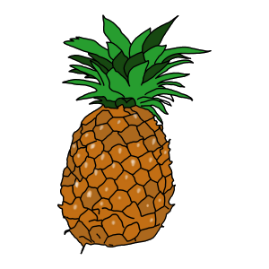

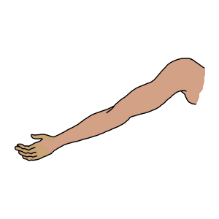

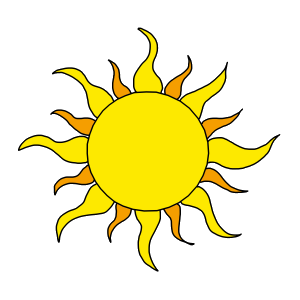


| 359 | 381 | 442 | 473 | 488 |
| --- | --- | --- | --- | --- |


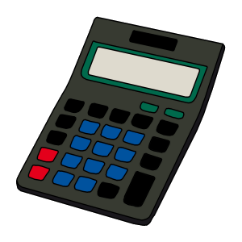

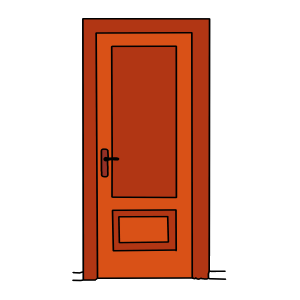

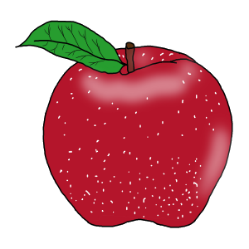

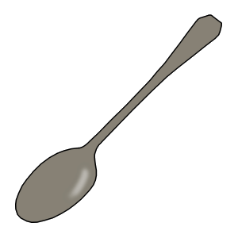

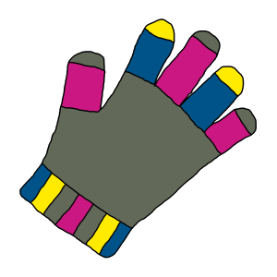


| 516 | 546 | 552 | 564 | 576 |
| --- | --- | --- | --- | --- |


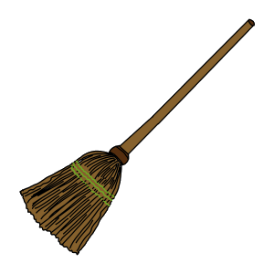

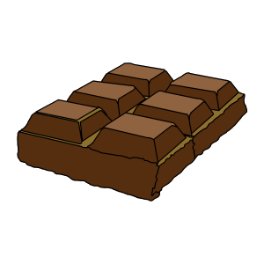

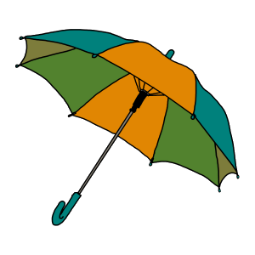

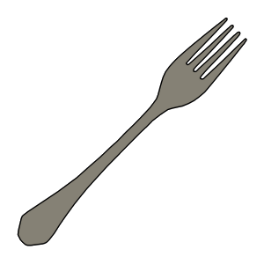

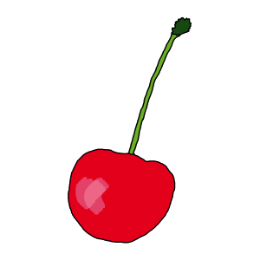


| 590 | 593 | 653 | 673 | 692 |
| --- | --- | --- | --- | --- |


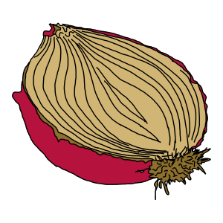

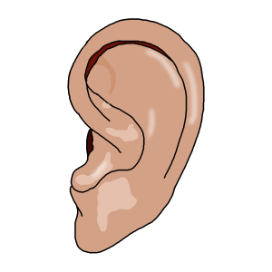

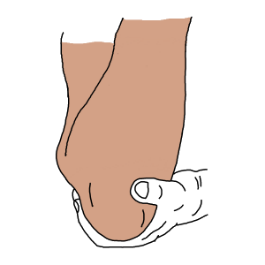

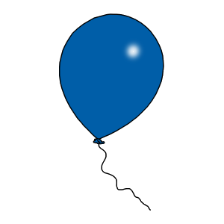

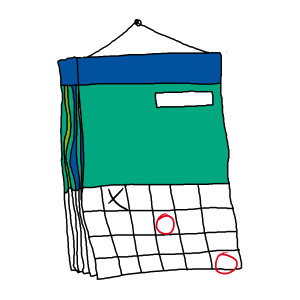


| 26 | 35 | 56 | 75 | 108 |
| --- | --- | --- | --- | --- |


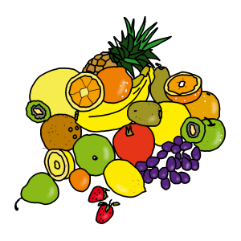

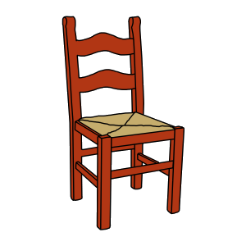

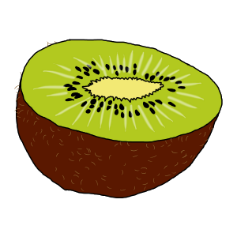

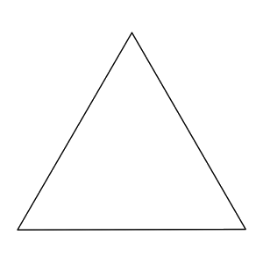

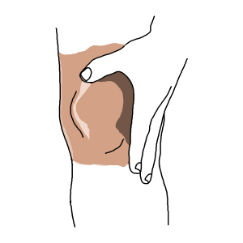


| 114 | 122 | 125 | 181 | 222 |
| --- | --- | --- | --- | --- |


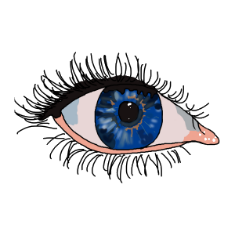

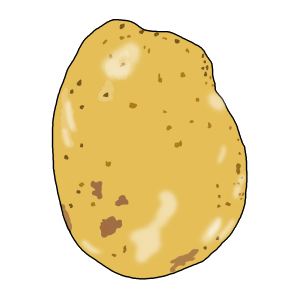

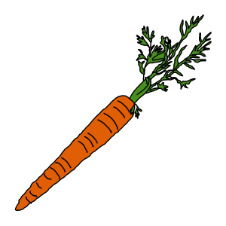

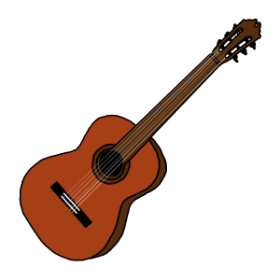

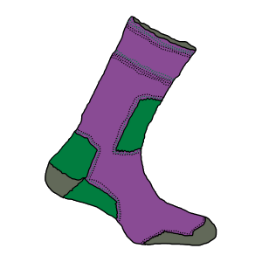


| 241 | 249 | 285 | 357 | 416 |
| --- | --- | --- | --- | --- |


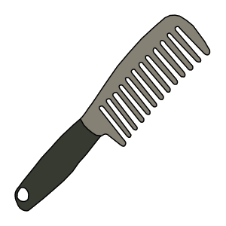

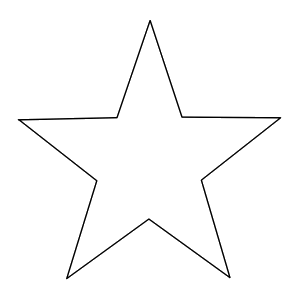

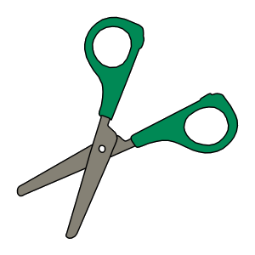

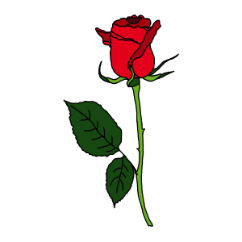

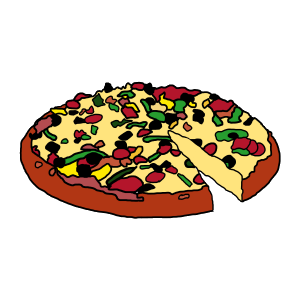


| 426 | 447 | 453 | 480 | 489 |
| --- | --- | --- | --- | --- |


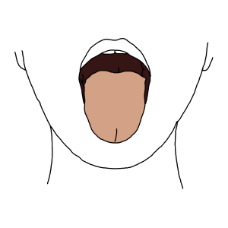

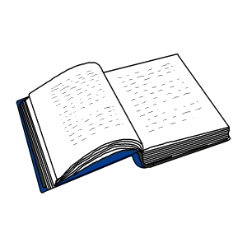

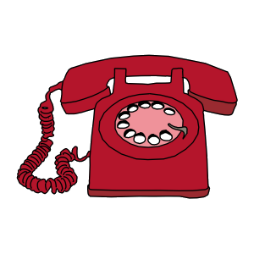

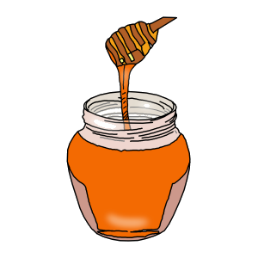

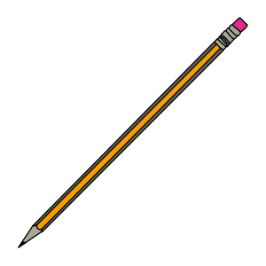


| 503 | 505 | 509 | 610 | 654 |
| --- | --- | --- | --- | --- |


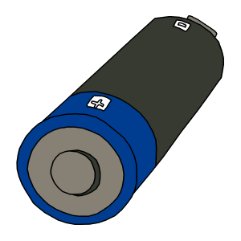

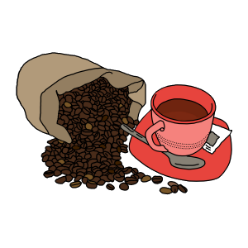

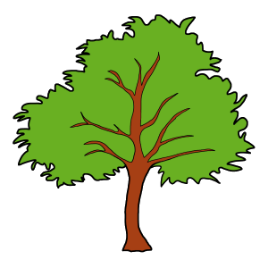

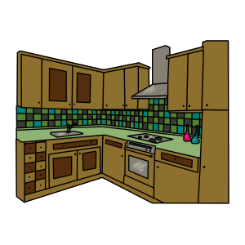

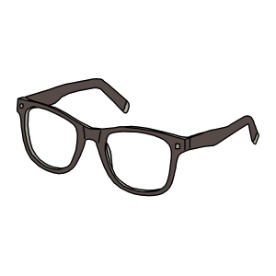


| 656 | 666 | 693 | 730 | 733 |
| --- | --- | --- | --- | --- |

Supplement: Supplementary file 1 [file mmc1.docx]
